# Linear and Nonlinear Intersubband Optical Properties of Direct Band Gap GeSn Quantum Dots

**DOI:** 10.3390/nano9010124

**Published:** 2019-01-19

**Authors:** Mourad Baira, Bassem Salem, Niyaz Ahmad Madhar, Bouraoui Ilahi

**Affiliations:** 1 Micro-Optoelectronic and Nanostructures Laboratory, Faculty of Sciences, University of Monastir, Monastir 5019, Tunisia; mourad.baira@isimm.rnu.tn; 2 CNRS, LTM, CEA-Leti, University Grenoble Alpes, 38054 Grenoble CEDEX 9, France; bassem.salem@cea.fr; 3 Department of Physics and Astronomy, College of Sciences, King Saud University, Riyadh 11451, Saudi Arabia; nmadhar@ksu.edu.sa

**Keywords:** GeSn, quantum dot, direct band gap, intersubband nonlinear optics, absorption coefficients, refractive index changes

## Abstract

Intersubband optical transitions, refractive index changes, and absorption coefficients are numerically driven for direct bandgap strained GeSn/Ge quantum dots. The linear, third-order nonlinear and total, absorption coefficients and refractive index changes are evaluated over useful dot sizes’ range ensuring *p*-like Γ-electron energy state to be lower than *s*-like L-electron energy state. The results show strong dependence of the total absorption coefficient and refractive index changes on the quantum dot sizes. The third order nonlinear contribution is found to be sensitive to the incident light intensity affecting both total absorption coefficient and refractive index changes, especially for larger dot sizes.

## 1. Introduction

A recent demonstration of direct bandgap GeSn alloys fully compatible with Complementary Metal Oxide Semiconductor (CMOS) technology [1,2,3,4] has generated intensive theoretical and experimental works aiming to explore their potentiality in the conception and implementation of optoelectronic devices [5,6,7]. Accordingly, optically pumped GeSn based laser diode, operating at low temperature, has already been demonstrated [7,8,9]. This has created real opportunity towards low-cost active optical devices monolithically integrable on Si substrates that may provide the missing part to Si photonic integrated circuits. Furthermore, all-optical switches and modulators are generally made from GaAs based semiconductor alloys [10,11], being challenging for integration on a Si platform. On the other hand, the weak nonlinear optical effects in Si based materials prohibits their effective on-chip integration [12]. Linear and nonlinear optical processes in nanostructures and specially quantum dot (QD) have generated an ongoing interest due to the possibility for the intersubband optical transition to occur with large dipole matrix element’s value leading to significant optical nonlinearities [13]. Accordingly, it is important to explore the linear and nonlinear optical processes in CMOS compatible low dimensional quantum structures. Thus, an emergent research activity has been dedicated to investigate GeSn based nanostructures, potentially interesting to improve the optoelectronic devices’ performance such as quantum wells [14], nanowires [15,16], nanorods [17], and Quantum dots [18,19,20,21,22].

This work aims to explore numerically, the impact of the QD size and incident light intensity on the linear and third order nonlinear refractive index changes (RIC) and absorption coefficients (AC) related to the intersubband optical transitions in GeSn QD. The reported results could serve as a roadmap for practical design and implementation of far IR optical devices.

## 2. Intersubband Transition Energies

**Figure 1:**
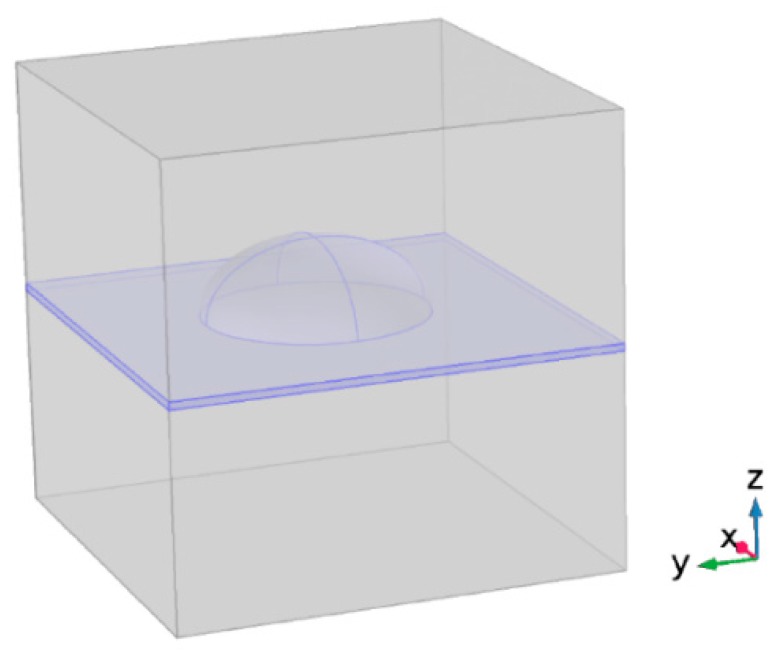
Schematic illustration of the investigated dome shaped quantum dot (QD).

The studied structure consists of dome shaped GeSn QD on top of one nm thick GeSn wetting layer surrounded by Ge matrix [23]. This QD has a typical design of conventional III-V compound [24] and element IV [25] based self-organized QDs as shown in Figure 1. We have considered the QD aspect ratio (defined as the dome height (H) to circular base diameter (D) ratio) to be 1/3 with a composition of Sn being 28%. The choice of this composition has been made based on recent advancements in the growth of GeSn material with high composition [17,26].

The electron’s confined energies and corresponding wave functions are numerically evaluated by solving the three-dimensional single band effective mass Schrodinger equation in Cartesian coordinates by finite elements method using COMSOL multiphysics software [27].

The Schrödinger equation is given by:

(1) $$-\frac{\hbar^{2}}{2}\nabla \left[\frac{1}{m^{*}\left(r\right)}\nabla \psi \left(r\right)\right]+V\left(r\right)\psi \left(r\right)=E\psi \left(r\right)$$

where *E* and ψ are the electron’s energy levels and wave function, m^*^ is the corresponding effective mass, r is the coordinate vector in Cartesian coordinates and V is the confining potential barrier. The Schrödinger equation has been solved for the electrons in Г and L bands taking into account the lattice mismatch induced strain. The calculation procedure and material parameters are detailed elsewhere [21,22].

**Figure 2:**
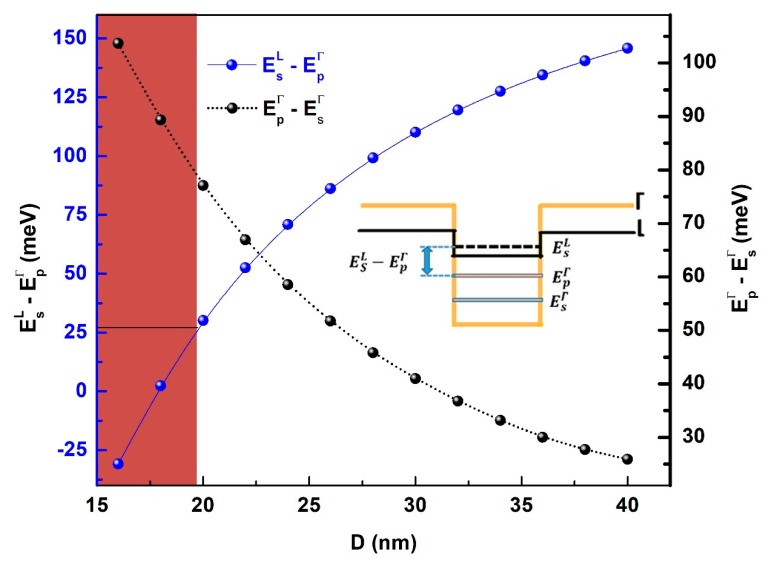
Intersubband transition energy $\left(E_{p}^{\Gamma }-E_{s}^{\Gamma }\right)$ and energy difference between *p*-like electron energy level in Г-valley and *s*-like electron energy levels in L valley $\left(E_{s}^{L}-E_{p}^{\Gamma }\right)$ as a function of the QD diameter.

Indeed, to warrant explicit involvement of the Г-electrons in the intersubband transition, the *p*-like electron energy level in Г-valley should be lower than the ground state electron confined energy in the L valley [22,28] as illustrated by the inset of the Figure 2. Since these energy levels are only dependent on the QD size, it is important to identify the efficient sizes range that allows satisfying this condition. The fulfillment of this requirement limits this study to the practically exploitable intersubband transitions.

Figure 2 shows the evolution the intersubband transition energy $\left(E_{p}^{\Gamma }-E_{s}^{\Gamma }\right)$ as well as the energy difference between the *p*-like electron energy level in Г-valley and the ground state electron energy in the L valley $\left(E_{s}^{L}-E_{p}^{\Gamma }\right)$ for QD diameters ranging from 16 to 40 nm. The QD sizes, where the mentioned condition is not applicable, are indicated by the red box in Figure 2. It is found that only QD diameters above 20 nm can be practically useful for efficient intersubband electron transitions. Indeed, the condition $E_{s}^{L}-E_{p}^{\Gamma }>26\;\mathrm{meV}$ avoids the loss of the electrons by thermal activation to the confined states in the L band. For the same raison, the upper size limit for the QD is also limited by maintaining the intersublevel energies higher than the thermal energy at room temperature. Accordingly, the interaband transition energies, in this case, can only be tuned between 26 and 78 meV.

The inset shows a simplified schematic representation of the QD conduction band at Γ and L points as well as the electron confined energy. The wetting layer contribution to the schematic band structure has been omitted for simplicity.

**Figure 3:**
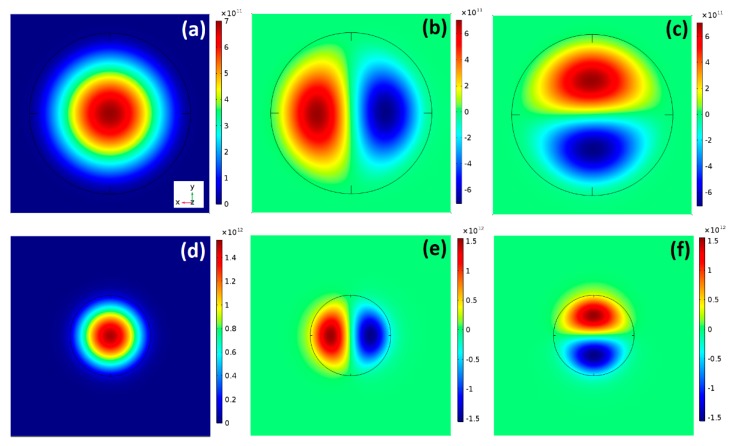
In plane-normalized s (**a**,**d**), *p*_x_ (**b**,**e**) and *p*_y_ (**c**,**f**) electron envelope wave function for a QD with diameter 40 nm and 20 nm respectively.

Figure 3 illustrates the *s*- and *p*-like Г electron envelope wave functions in the *XY* plane for the smallest (D = 20 nm) and the largest (D = 40 nm) QD size. The obtained results follow those reported for lens-shaped InAs/GaAs quantum dots [29] and indicate good electron confinement over the exploitable QD sizes range.

The *p* states, demonstrated to be fully in-plane polarized [30,31], are twofold degenerated due to the cylindrical symmetry [32,33]. Furthermore, in the case of ideal QD, free of alloy and/or shape fluctuation having one electron per QD, these states are expected [29] to be oriented towards the crystallographic directions [110] and $\left[1\bar{1}0\right]$. Consequently, by choosing the *X* and *Y*-axis orientation along these crystallographic directions, the *p*-like states can be identified as $p_{x}$ and $p_{y}$. The two *p* states are equivalent (can be generated from each other by a rotation of π/2 around *z*-axis). A selection rule, for the in-plane polarized light generated intersubband transition from *p* to *s* shell, can be established depending on the light polarization direction. Indeed, if the light is polarized along *X*, only the transition from the $p_{x}$ state will be allowed [30,31].

## 3. Linear and Nonlinear Optical Properties

The analytical expression of the linear ($\chi^{\left(1\right)}$) and third order nonlinear ($\chi^{\left(3\right)}$) optical susceptibilities obtained by considering the QD as a two-level system are given by: [34,35,36]

(2) $$\chi^{\left(1\right)}\left(\omega \right)=\frac{\sigma }{\varepsilon_{0}\hbar }\frac{{\left|M_{fi}\right|}^{2}}{\left(\omega_{fi}-\omega -j\Gamma \right)}$$

(3) $$\chi^{\left(3\right)}\left(\omega \right)=\frac{-\sigma {\left|M_{fi}\right|}^{2}{\left|F\right|}^{2}}{\varepsilon_{0}\hbar^{3}\left(\omega_{fi}-\omega -j\Gamma \right)}\times \left[\frac{4{\left|M_{fi}\right|}^{2}}{\left({\left(\omega_{fi}-\omega \right)}^{2}+\Gamma^{2}\right)}-\frac{{\left(M_{ff}-M_{ii}\right)}^{2}}{\left(\omega_{fi}-j\Gamma \right)\left(\omega_{fi}-\omega -j\Gamma \right)}\right]$$

*F* is the electrical field intensity associated to the incident light intensity by the following relation: $I=\frac{2n_{r}}{\mu c}{\left|F\right|}^{2}$. Where $n_{r}$, μ and c are respectively, the QD materials refractive index, the permeability, and the free space speed of light. The GeSn refractive index value is derived from those of its constituent material by linear interpolation ($n_{r}=4.051$ for Ge 5.791 for α-Sn [37]).

σ represents the carrier’s density turning out to be the inverse of the QD volume in the present case assuming one electron per QD [36]. $\varepsilon_{0}$ is the dielectric permittivity of free space, ω is the angular frequency, $\omega_{fi}$ is the transition angular frequency related to the intersubband transition energy by $\frac{E_{p}^{\Gamma }-E_{s}^{\Gamma }}{\hbar }$. Γ is the relaxation rate taken to be $\Gamma =\frac{1}{\tau }$, where τ is the relaxation time taken to be 0.1 ps [38].

$M_{fi}=\langle \psi_{f}|ex|\psi_{i}\rangle$ represents the dipole moment for light polarization along *X* direction and the subscripts *f* and *i* denote the final and initial states referring to the *p*_x_- and *s*-like electron states in the QD’s Γ valley.

The real part of the total susceptibility χ(ω) is associated to the total refractive index change as follows [31]:

(4) $$\frac{\delta n\left(\omega \right)}{n_{r}}=Re\left(\frac{\chi \left(\omega \right)}{2n_{r}^{2}}\right)=\frac{\delta n^{\left(1\right)}\left(\omega \right)}{n_{r}}+\frac{\delta n^{\left(3\right)}\left(\omega \right)}{n_{r}}$$

where $\frac{\delta n^{\left(1\right)}\left(\omega \right)}{n_{r}}$ and $\frac{\delta n^{\left(3\right)}\left(\omega \right)}{n_{r}}$ are respectively the linear and third order nonlinear contribution to the refractive index change. Their analytical expression is given by the Equations (5) and (6).

(5) $$\frac{\delta n^{\left(1\right)}\left(\omega \right)}{n_{r}}=\frac{\sigma {\left|M_{fi}\right|}^{2}}{2n_{r}^{2}\varepsilon_{0}\hbar }\frac{\omega_{fi}-\omega }{\left[{\left(\omega_{fi}-\omega \right)}^{2}+\Gamma^{2}\right]}$$

(6) $$\begin{array}{c} \frac{\delta n^{\left(3\right)}\left(\omega ,\;I\right)}{n_{r}}=\frac{-\mu \sigma I}{4n_{r}^{3}\varepsilon_{0}\hbar^{3}}\frac{{\left|M_{fi}\right|}^{2}}{{\left[{\left(\omega_{fi}-\omega \right)}^{2}+\Gamma^{2}\right]}^{2}}\times [4\left(\omega_{fi}-\omega \right){\left|M_{fi}\right|}^{2}-\frac{{\left(M_{ff}-M_{ii}\right)}^{2}}{\omega_{fi}^{2}+\Gamma^{2}}\{\left(\omega_{fi}-\omega \right)\times \\ \left[\omega_{fi}\left(\omega_{fi}-\omega \right)-\Gamma^{2}\right]-\Gamma^{2}\left(2\omega_{fi}-\omega \right)\}] \end{array}$$

**Figure 4:**
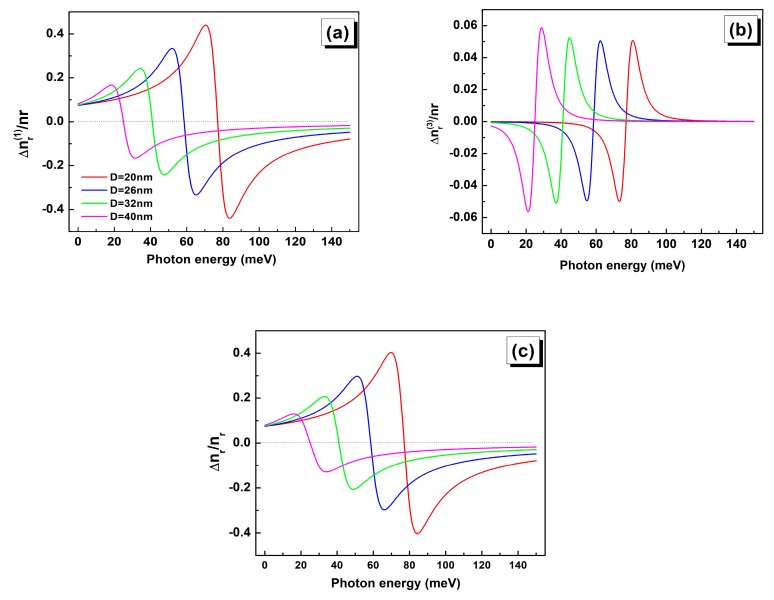
Calculated linear (**a**), 3rd order nonlinear (**b**) and total refractive index changes (RIC) (**c**) as a function of the photon energy for different QD sizes: D = 20 nm (red), D = 26 nm (blue), D = 32 nm (green), D = 40 nm (pink).

The Figure 4, shows the calculated linear, third order nonlinear and total RIC for different QD sizes (D = 20, 26, 32 and 40 nm) as a function of the photon energy for an incident light intensity of 0.4 $\mathrm{MW}\cdot {\mathrm{cm}}^{-2}$.

The linear RIC is found to decrease with increasing the QD size. Meanwhile, the nonlinear RIC slightly increases in magnitude while being opposite in sign to the linear RIC. Consequently, the total RIC is further reduced as the QD size increases when compared to its linear part. The observed shift of the linear, nonlinear, and total RIC towards lower energy with increasing the QD size is an obvious consequence of the intersubband transition energy decrease. This result clearly indicates that the total RIC strongly depends on the QD volume change. Hence, for accurate evaluation of the refraction index change, it is recommended to take into account the third order nonlinear term for larger QD sizes. Additionally, the strong reduction in the total RIC with increasing QD size is mainly due to the electron density evolving as the inverse of the QD volume. To obtain larger RIC from bigger QD, the increase of σ can be considered as a viable option within the appropriate incident light intensity.

The optical absorption coefficients related to the intersubband transition are also an important parameter that needs to be evaluated for this novel QD system. Indeed, the absorption coefficient (AC) can be numerically driven from the imaginary part of the optical susceptibility ensuing the following equation [31,32]:

(7) $$\alpha \left(\omega \right)=\omega \sqrt{\frac{\mu }{\varepsilon_{r}}}Im\left[\varepsilon_{0}\chi \left(\omega \right)\right]=\alpha^{\left(1\right)}\left(\omega \right)+\alpha^{\left(3\right)}\left(\omega ,I\right)$$

where the linear absorption coefficient takes the following expression:

(8) $$\alpha^{\left(1\right)}\left(\omega \right)=\frac{\omega }{\hbar }\sqrt{\frac{\mu }{\varepsilon_{r}}}\frac{\sigma {\left|M_{fi}\right|}^{2}\Gamma }{\left[{\left(\omega_{fi}-\omega \right)}^{2}+\Gamma^{2}\right]}$$

In addition, the third order nonlinear AC is described by the following equation:

(9) $$\begin{array}{cc} \alpha^{\left(3\right)}\left(\omega ,I\right)= & \left(\frac{-\omega \sigma I}{2\varepsilon_{0}n_{r}c\hbar^{3}}\right)\sqrt{\frac{\mu }{\varepsilon_{r}}}\times \frac{{\left|M_{fi}\right|}^{2}\Gamma }{{\left[{\left(\omega_{fi}-\omega \right)}^{2}+\Gamma^{2}\right]}^{2}}\\ & \;\times \left[4{\left|M_{fi}\right|}^{2}-\frac{{\left(M_{ff}-M_{ii}\right)}^{2}\left[3\omega_{fi}^{2}-4\omega_{fi}\omega +(\omega^{2}-\Gamma^{2}\right]}{\omega_{fi}^{2}+\Gamma^{2}}\right] \end{array}$$

**Figure 5:**
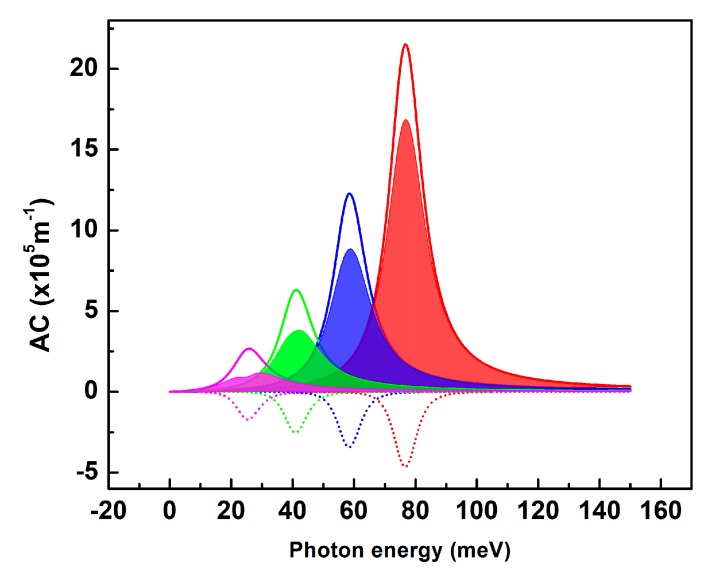
Calculated linear (solid lines), 3rd order nonlinear (dotted lines) and total absorption coefficient (AC) (filled area curves) as a function of photon energy for a selection of QD sizes: D = 20 nm (red), D = 26 nm (blue), D = 32 nm (green), D = 40 nm (pink).

The linear, third order nonlinear and total AC are plotted in the Figure 5 as a function of the photon energy for different values of the QD diameter. The maximum peak matches the intersubband transition energy leading to the observed displacement towards lower energies when the QD size increases. Furthermore, the linear AC is reduced with increasing QD size inducing the decrease in the resonance peak’s intensity from $22\times 10^{5}{\;m}^{-1}$ for the smallest QD size down to $2.7\times 10^{5}{\;m}^{-1}$ for the larger one. In the counterpart, the third order nonlinear AC shows a negative resonance peak intensity slowly decreasing in absolute value when the QD size increases. Consequently, the variation of the total AC is strongly influenced by the increase in the QD size [39].

**Figure 6:**
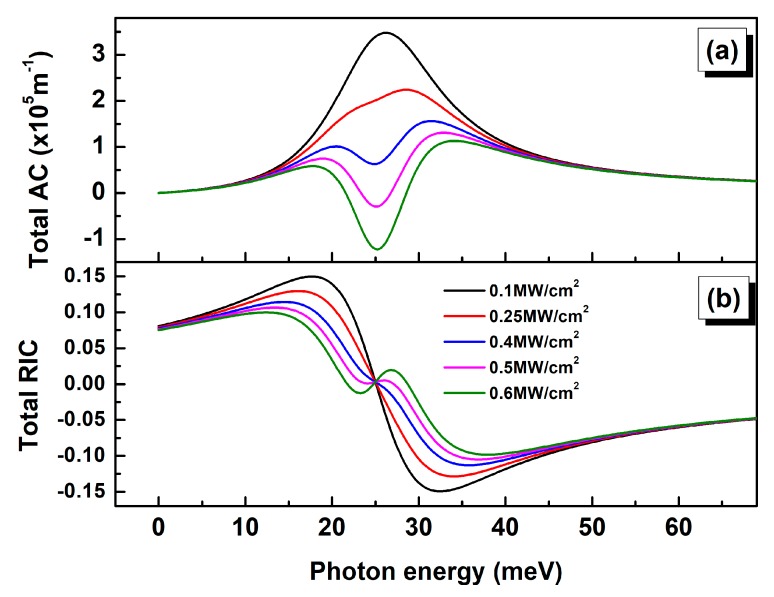
Incident optical intensity dependence of total optical AC (**a**) and RIC (**b**) as a function of the photon energy for D = 40 nm.

We notice a relative saturation in the total AC for the 40 nm diameter QD as the third order nonlinear peaks intensity exceeds half of the linear one. Indeed, the third order nonlinear term’s magnitude is strongly dependent on the incident light intensity (I). According to Equations (6) and (9), increasing I results in an increase in both AC and RIC nonlinear terms. Since the linear and third order nonlinear terms are of opposite sign, the total AC and RIC will consequently be strongly affected. As bigger QD are more sensitive to the nonlinear contribution, we have numerically evaluated the total AC and RIC for D = 40 nm with incident light intensity ranging from 0.1 to 0.6 $\mathrm{MW}\cdot {\mathrm{cm}}^{-2}$ (Figure 6).

The calculation results show that the magnitude of the total absorption coefficient decreases with increasing the incident light intensity. As shown by the Figure 6a, the resonance peak’s intensity saturates for I higher than 0.2 $\mathrm{MW}\cdot {\mathrm{cm}}^{-2}$. Similarly, increasing the incident optical intensity leads to an overall reduction in the total RIC (Figure 6b). Our calculation shows that the optical AC and RIC for GeSn self-assembled QD appears to be strongly dependent on both size and incident light intensity. Accordingly, for bigger QD sizes the nonlinear effects are dominant factors, especially for relatively high incident intensity.

## 4. Conclusions

In this paper, we have calculated the linear, third order nonlinear and total AC and RIC as a function of the GeSn QD size and incident light intensity. The QD size has been delimited by the specific directness parameter that ensures the intersubband transition to occur within the Г band. The third order nonlinear contribution to the AC and refractive index is found to be strongly dependent on the QD size and incident light intensity. The results could help in designing and implementing CMOS compatible optical devices for photonic integrated circuits.

## Abbreviations

QD	Quantum Dots
AC	absorption coefficient
RIC	refractive index change
CMO	Complementary Metal Oxide Semiconductor
